# Biotics supplementation of infant formula: Lessons for the development and testing of infant formulas?

**DOI:** 10.1002/jpn3.70234

**Published:** 2025-11-06

**Authors:** Mary S. Fewtrell

**Affiliations:** ^1^ Population, Policy & Practice Research and Teaching Department UCL Great Ormond Street Institute of Child Health London UK

**Keywords:** bioactive components, breast milk substitute, breastfeeding, regulation

Lactation evolved over millions of years to provide nutrition for mammalian infants and to act as a regulatory signal between mother and offspring, allowing the mother to transmit information about her current environment as well as her previous experiences which can shape the infant's growth and development.^1^ Milk is a complex and highly variable fluid containing nutrients but also numerous bioactive components including hormones, growth factors, biotics, microRNAs (miRNAs), as well as cells and its own microbiome. While the function and mechanism of action of many components is still poorly understood, it is thought that they may explain some of the differences in health and developmental outcomes observed between individuals who receive breast milk versus those who receive breast milk substitutes (infant formulas).

As well as being the biological norm, breastfeeding is an important public health intervention, with health benefits for mother and infant.^2^ Exclusive breastfeeding is recommended for the first 6 months of life by the World Health Organization ^3^ and for at least the first 4 months of life by the European Society for Pediatric Gastroenterology, Hepatology and Nutrition (ESPGHAN).^4^ However, despite many initiatives, compliance with recommendations is poor in most settings—particularly in higher‐income countries—and many healthy infants are breast‐fed for less than 6 months or not at all. In this situation, the only safe alternative is an infant formula.^5^ These products may be the sole source of nutrition during a vulnerable period of postnatal life when infants are growing and developing rapidly. Hence, the design and composition of infant formulas must be based on rigorous science with robust safety and efficacy testing. The aim is not to mimic the composition of human milk—which would be impossible—but to reduce the gap in health and developmental outcomes between infants receiving breast milk and those receiving infant formula.

Over the last 40 years, infant formulas have been modified in many ways. This includes adaptation of macro‐ and micronutrient content but also the addition of potential bioactive components that are present in human milk but not present (or not in the same quantity or structure) in formulas which are largely derived from cows' milk. The hope has been that adding these components to an infant formula may help reduce the gap in outcomes between breast‐fed versus formula‐fed infants.

One group of bioactive components which has received increasing research focus over the past 20 years is the biotics—probiotics, prebiotics, human‐identical milk oligosaccharides, synbiotics, and postbiotics. The rationale for adding biotics to infant formula is that there is evidence suggesting they can influence gastrointestinal microbiota composition, stimulating the development of a bifidogenic microbiota and decreasing amounts of possible pathogens, which in turn may have an impact on both shorter‐ and longer‐term health outcomes.

The manuscript from the European Society for Pediatric Gastroenterology, Hepatology, and Nutrition Special Interest Group on Gut Microbiota & Modifications (ESPGHAN SIG‐GMM) on the supplementation of infant formulas with biotics ^6^ addresses two important and timely questions. First, which clinically relevant benefits have been demonstrated and second, should biotics be added to infant formula and, if yes, which specific biotic and for which indications? The review summarizes the findings of five separate technical reviews^7^, ^8^, ^9^, ^10^, ^11^ which used a rigorous systematic approach to identify and synthesize the evidence on safety and efficacy of formula supplementation, a modified Delphi process to derive statements and recommendations followed by a public consultation via the ESPGHAN website.

The SIG concludes that “infant formulas supplemented with one or more of the biotics evaluated so far fed to healthy infants do not raise safety concerns regarding growth, tolerance, and adverse effects.” However, with regard to efficacy they concluded that for most biotics evaluated so far, no recommendations could be made either “in favor” or “against” supplementation; the exception being a weak recommendation in favor of some prebiotics (scGOS/lcFOS) because of observed stool softening effects.

So—where does this conclusion leave us from a practical perspective and for future research and development? The SIG highlighted the variability in study designs, intervention types (duration, amount, and composition) and measured outcomes, which limited their ability to draw conclusions—in particular that recommendations were formulated only if at least two well‐designed randomized controlled trials (RCTs) were available and there were rarely two studies with the same biotic supplementation and a similar design. Ultimately, they stated that their conclusion may reflect the limited data on specific biotics and outcomes because of the heterogeneity in the RCTs rather than an actual lack of effect; we are well aware that absence of evidence does not equate to evidence of no effect.

The conclusions of the SIG paper with respect to biotics has parallels with the addition of other components to infant formulas—notably experience over many years with long‐chain polyunsaturated fatty acid (LCPUFAs) supplementation. The clear biological importance of LCPUFAs, their presence in breast milk but not in older infant formulas and the gap in neurodevelopmental outcomes between breast and formula fed infants led to the plausible hypothesis that supplementing infant formulas might narrow the outcome gap. In practice, however, despite numerous RCTs and evidence of the biochemical efficacy of supplemented formulas, clinically relevant effects on outcome have not been proven.^12^, ^13^ Suggested explanations for the lack of anticipated effects include many of those identified by the SIG in their biotics review, including marked heterogeneity between trials in design, population, and protocol but perhaps most importantly in the intervention itself, since LCPUFAs have been added to formulas in different combinations, quantities, and from very different sources. It is also plausible that components added to an infant formula may not function in the same way as they do in the complex matrix of human milk. In this context, the discussion and recommendations of the SIG regarding biotics supplementation of infant formulas could arguably be widened to include infant formula modifications more generally.

Adding additional components to formulas may increase costs. Furthermore, as stated by the European Food Safety Authority in its Scientific Opinion on the essential composition of infant and follow‐on formulae,^5^ “nutrients and substances should be added to formulae for infants only in amounts that serve a nutritional or other benefit. The addition in amounts higher than those serving a benefit or the inclusion of unnecessary substances in formulae may put a burden on the infant′s metabolism and/or on other physiological functions, as substances which are not used or stored have to be excreted.” However, whilst seemingly uncontroversial, this statement does not address the process and criteria for establishing that a substance has “proven benefits” in the real world. How can we improve research on infant formula modifications to increase the likelihood of detecting effects if present or excluding them if not?

The SIG makes several recommendations to address this. They highlight the need for multiple independent studies—ideally at least two independent randomized controlled trials of high quality with sufficient power and with the same design and biotic. However, it is not clear how this would happen in the current environment where infant formula research is largely industry‐driven, led and funded, and companies inevitably set their priorities based on commercial as well as scientific priorities. Fully independent research with no industry involvement or sponsorship may not be feasible due to high costs and logistical challenges and there is the risk that without the involvement and investment of industry, improvements in infant formula composition would be slower or may cease altogether. The SIG recommends that collaboration among regulatory bodies, academia, and industry is crucial to conduct unbiased studies. As they state, “by pooling resources and expertise, regulatory bodies, academia, and industry can ensure rigorous research that avoids conflicts of interest as well as identify and prioritize scientifically and clinically relevant outcomes addressing infant health rather than industry‐funded goals.”

There is no question that breastfeeding is the gold standard which must be promoted, supported, and protected. However, optimizing the design of breast milk substitutes, the only safe alternative when breast milk is not available, is also a public health imperative. Achieving greater collaboration between regulatory bodies, academia, and industry—including input from the public—will not be straightforward, especially given the current often adversarial environment surrounding infant nutrition. However, without improvement in our approach to infant formula research and regulation, there is a risk that in 10 years' time we may be drawing the same conclusions about the safety and efficacy of supplementation of infant formula with another bioactive component.

## CONFLICT OF INTEREST STATEMENT

The author declares no conflicts of interest.
